# High cases of submicroscopic *Plasmodium falciparum* infections in a suburban population of Lagos, Nigeria

**DOI:** 10.1186/s12936-019-3073-7

**Published:** 2019-12-19

**Authors:** Florence A. Umunnakwe, Emmanuel T. Idowu, Olusola Ajibaye, Blessed Etoketim, Samuel Akindele, Aminat O. Shokunbi, Olubunmi A. Otubanjo, Gordon A. Awandare, Alfred Amambua-Ngwa, Kolapo M. Oyebola

**Affiliations:** 1 Parasitology and Bioinformatics Unit, Department of Zoology, Faculty of Science, University of Lagos, Akoka, Lagos Nigeria; 2 Nigerian Institute of Medical Research, Yaba, Lagos, Nigeria; 3 Medical Research Council at the London School of Hygiene and Tropical Medicine, Fajara, The Gambia; 4 West African Centre for Cell Biology of Infectious Pathogens, University of Ghana, Legon, Accra, Ghana

**Keywords:** Asymptomatic malaria, Rapid diagnostic test, Microscopy, *var*ATS, *Plasmodium falciparum*, Nigeria, qPCR

## Abstract

**Background:**

Asymptomatic malaria parasites are significant sources of infections for onward malaria transmission. Conventional tools for malaria diagnosis such as microscopy and rapid diagnostic test kits (RDT) have relatively low sensitivity, hence the need for alternative tools for active screening of such low-density infections.

**Methods:**

This study tested *var* acidic terminal sequence-based (*var*ATS) quantitative polymerase chain reaction (qPCR) for screening asymptomatic *Plasmodium falciparum* infections among dwellers of a sub-urban community in Lagos, Nigeria. Clinically healthy participants were screened for malaria using microscopy, RDT and *var*ATS qPCR techniques. Participants were stratified into three age groups: 1–5, 6–14 and > 14 years old.

**Results:**

Of the 316 participants screened for asymptomatic malaria infection, 78 (24.68%) were positive by microscopy, 99 (31.33%) were positive by RDT and 112 (35.44%) by *var*ATS qPCR. Participants aged 6–14 years had the highest prevalence of asymptomatic malaria, with geometric means of ~ 116 parasites/µL and ~ 6689 parasites/µL as detected by microscopy and *var*ATS, respectively.

**Conclusion:**

This study has revealed high prevalence of asymptomatic malaria in the study population, with *var*ATS detecting additional sub-microscopic infections. The highest concentration of asymptomatic malaria was observed among school-age children between 6 and 14 years old. A large-scale screening to identify other potential hotspots of asymptomatic parasites in the country is recommended.

## Background

Malaria remains a major cause of morbidity and mortality in sub-Saharan Africa. The prevalence of malaria has remained static in recent years unlike the success recorded in early 2000s [1]. Consequently, there is a need for localized and country-specific interventions, particularly prompt diagnosis of infections and treatment to control transmission. Nigeria currently contributes approximately one-third of the global burden of malaria [1]. Control efforts in the country rely principally on passive diagnosis at health facilities by clinical examination or by parasitological confirmation [2]. However, the recent transition in malaria research priorities from control to elimination emphasizes the need to monitor both clinical and asymptomatic infections [3]. While case detection of clinical malaria has been largely successful [4, 5], confirmation of asymptomatic infections, which are usually below microscopy-detectable levels and are rarely treated, remains a major challenge [6]. As a major source of parasites for local mosquito vectors, undetected asymptomatic infections contribute to the persistence of malaria transmission [7]. These asymptomatic parasites have also been linked with chronic anaemia and co-infections with invasive bacteria [8] .

Although there have been some success stories of malaria reduction following rapid diagnostic test kits (RDTs) and microscopy-based diagnosis [9, 10], both tools have limited sensitivity in detecting sub-clinical infections [11, 12]. It is believed that better malaria control or elimination outcomes would be achieved if low-density parasitaemias were detected [13, 14]. Moreover, the introduction of conventional polymerase chain reaction (PCR) tools in estimating the burden of asymptomatic malaria has achieved only little improvement at a threshold of 0.05–10 parasites/µL [15, 16]. Since parasitaemia of 0.05/μL of blood corresponds to as high as approximately 100,000 parasites in the body [17], such undetectable ultra-low infections may sustain malaria transmission.

A relatively recent technique for the ultra-sensitive detection of low parasitaemia has so far yielded promising outcomes [18]. This procedure involves the amplification of the *var* gene family present in the sub-telomere of the parasite. Each parasite isolate comprises about 50–150 *var* genes, which possess acidic terminal sequences (ATS) with well-conserved domains [18]. PCR targeting *var*ATS of the parasite has a high sensitivity with a limit of detection of 0.03 parasites/μL blood [18]. While microscopy, RDTs and conventional PCR tools have been extensively evaluated for asymptomatic malaria diagnosis in Nigeria [19–22], there are no data on the performance and effectiveness of *var*ATS quantitative PCR (*var*ATS qPCR) for diagnosing asymptomatic *Plasmodium falciparum* infections in the country. This study evaluated the effectiveness of *var*ATS qPCR against conventional microscopy and RDTs for the assessment of asymptomatic *P. falciparum* carriage among sub-urban settlers in Lagos, Nigeria.

## Methods

### Study design and sample collection

**Fig. 1:**
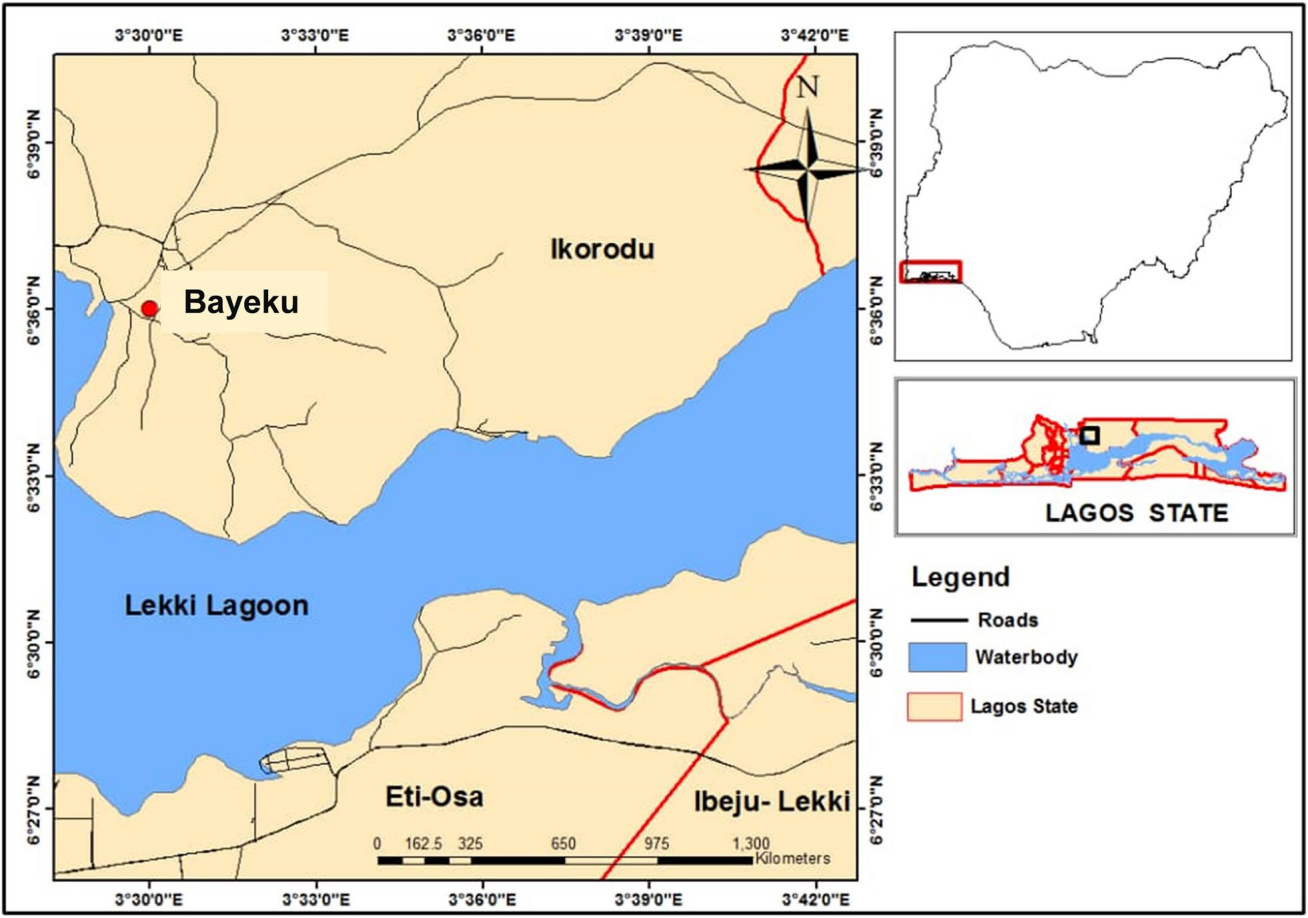
Map of Nigeria (inset) showing the location of study area

A cross-sectional, community-based study was carried out in July 2018 to screen residents of Bayeku for asymptomatic malaria. Bayeku is a rural community in Ikorodu Local Government, an outskirt of Lagos, Nigeria. Malaria is meso-endemic in Lagos, with peak transmission during the rainy season between April and September of every year [23, 24]. The geographical coordinates of Bayeku are 6° 35′ 60′′ N and 3° 30′ 0′′ E (Fig. 1). Following mobilization and advocacy by community representatives, screening was conducted at the community town hall within the palace of the traditional ruler. Blood samples were collected from a finger prick for microscopy and RDT performance. For molecular analysis, blood was spotted on filter paper (Whatmann 3MM General Electric Healthcare Co., UK). The blood spots were left on the bench to dry after which they were put in individual ziplock plastic bags containing desiccant and stored at room temperature (< 30 °C). Participants were visibly healthy individuals who had been resident in the community for more than 2 years and were without a history of fever or symptoms suggestive of malaria in the preceding 2 days. Exclusion criteria in this investigation were non-consenting individuals, patients who received anti-malarial therapy in the preceding 4 weeks before sampling, pregnant women as well as children under 1 year old, to preclude the influence of maternally inherited immunity [25]. Asymptomatic (or sub-clinical) malaria was defined as the presence of malaria parasites in a patient who showed no clinical evidence of infection at the time of diagnosis [8]. Participants were considered asymptomatic when the body temperature was < 37.5 °C at the time of blood collection and the participant reported no fever within the previous 2 days.

### Rapid diagnostic testing (RDT) and microscopy

Blood samples were tested for malaria parasites using CareStart™ Malaria HRP2 (*P. falciparum*; CAT NO: G0141, Access Bio Inc., USA). Thick and thin blood films were prepared on a glass slide for parasite identification and speciation using Giemsa technique. The slides were stained and viewed using 100× oil immersion objective lens of a compound microscope. At least 100 high power fields were examined before a thick smear was reported as negative. For positive slides, parasite species and stages were assessed and parasitaemia (parasite density) was determined by counting only the asexual stages against 300 white blood cells (WBC) and then multiplying by 25, assuming the mean total WBC count of individuals is 7500 cells/μL of blood [17]. Slides were blindly read by two independent level 1 microscopists. Positive/negative cases were called only after confirmation by both microscopists. Microscopy-based estimates of parasite density were calculated as the average of the values that were within the margin of between-reader difference. Two readings were considered discrepant if their difference was outside the 95% range of the limits of agreement of previous paired readings [26]. The level of parasitaemia was recorded as low (< 1000 parasites/μL of blood), moderate (1000–9999 parasites/μL of blood), and severe (≥ 10,000 parasites/μL of blood) [27].

### *var*ATS qPCR diagnosis of asymptomatic malaria

*Plasmodium falciparum* DNA was extracted from dried blood spots using the QiaAmp DNA minikit (Qiagen, Germany). The *var* gene acidic terminal sequence (*var*ATS) quantitative PCR was used to detect multi-copy genomic sequences of low-density infections [18]. The primer/probe sequences and the cycling conditions are described in Table 1. Briefly, 1 μL of PCR water, 10 μL of 2 × Taqman Universal PCR Mastermix (Applied Biosystems, New Jersey, USA), 1.6 μL of 10uM forward and reverse primers, 0.8 μL of 10 μM probe and 5 μL of parasite DNA were vortexed and run on CFX 96 Touch™ Real-Time System (Bio-Rad Laboratories, CA, USA). The turnaround time was approximately 3 h. The starting quantity (SQ) values of the parasite samples were estimated against laboratory grown *P. falciparum* 3D7 standard control (with medonic read of 3.74 × 106 erythrocytes/µL and thin film parasitaemia of 1197 parasites/µL of blood). The serial dilution procedure is described in Additional file 3: Sheet S1.

**Table 1:** Primer sequences and qPCR conditions for *var*ATS assay

Oligonucleotide sequences	
Primer-fw (5′–3′)	CCCATACACAACCAATTGGA
Primer-rev (5′–3′)	TTCGCACATATCTCTATGTCTATCT
Probe (5′–3′)	6-FAM-TTTTCCATAAATGGT-NFQ-MGB
qPCR reaction conditions (final concentration in qPCR mix)	
Total volume (μL)	20
DNA volume (μL)	5
TaqMan^®^ Gene Expression Mastermix	1×
Primer (each)	800 nM
Probe	400 nM
qPCR cycling conditions	
Pre-incubation	2 min–50 °C
Initial denaturation	10 min–95 °C
Denaturation	15 s–95 °C
Annealing and elongation	1 min–55 °C
Number of cycles	45
Standard material for quantification	gDNA of parasite dilution row
Platform	Real-Time PCR System (Applied Biosystems)

### Statistical analysis

Participants were stratified into three age categories: 1–5 years, 6–14 years and > 14 years. Data from this study were coded, entered and analysed in GraphPad Prism 8. The difference in parasite densities between age groups was analysed using Chi-square (χ^2^) test and P-value < 0.05 was considered statistically significant. Student’s t-test was used for comparison of means. Kappa (K) coefficient was used to test the measure of agreement between RDT and *var*ATS as well as microscopy vs *var*ATS. K values < 0.20, 0.21–0.40, 0.41–0.60, 0.61–0.80, and 0.8–1.0 depicted poor, fair, moderate, good, and very good strengths of agreement, respectively [28].

## Results

A total of 316 asymptomatic participants were screened for *P. falciparum* out of which 125 (39.56%) were positive by at least one of microscopy, RDT and *var*ATS diagnostics. The demographic profile of participants is summarized in Table 2. Briefly, the ages of the participants ranged between 1 and 100 years with mean age = 23.15 years. Individuals 15 years and above formed the majority of participants with 153 (48.4%), this was followed by age group 6–14 years and 1–5 years with 96 (30.4%) and 67 (21.2%), respectively (Table 2).

**Table 2:** Demographic characteristics of participants

Variable	Attribute	Number (%)
Age group (years)	1–5	67 (21.2)
	6–14	96 (30.4)
	> 14	153 (48.4)
Gender	Female	201 (63.7)
	Male	115 (36.3)
Total		316

**Fig. 2:**
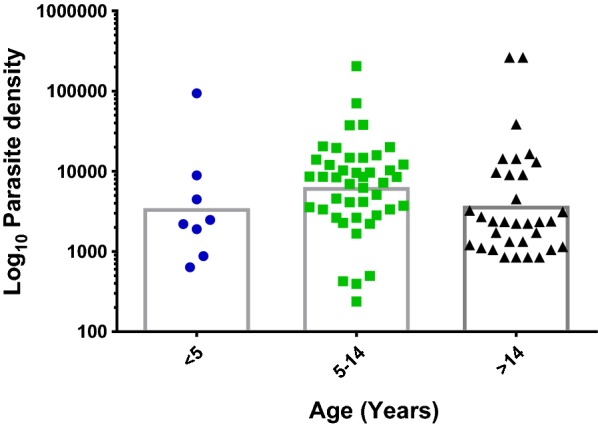
Age groups of participants and parasite density by *var*ATS (error bars showing geomean with 95% CI)

Microscopy/RDT/*var*ATS PCR counts were used to ascertain the prevalence of asymptomatic malaria in the study population. Prevalence of asymptomatic infections was 78 (24.68%) and 99 (31.33%) for microscopy and RDT, respectively, while *var*ATS qPCR detected additional 10% sub-microscopic infections in the population 112 (35.44%). The geometric mean parasite density by *var*ATS was higher in male participants (5409.86/µL) (Additional file 2: Tables S1, S2). When stratified by age groups (1–5, 6–14 and > 14 years), parasite densities were low in children younger than 5 (3566.28/µL). Participants aged 6–14 years had the highest cases and density of asymptomatic parasitaemia using *var*ATS qPCR with geometric mean of 6688.91/µL (Additional file 2: Table S3; Fig. 2).

**Fig. 3:**
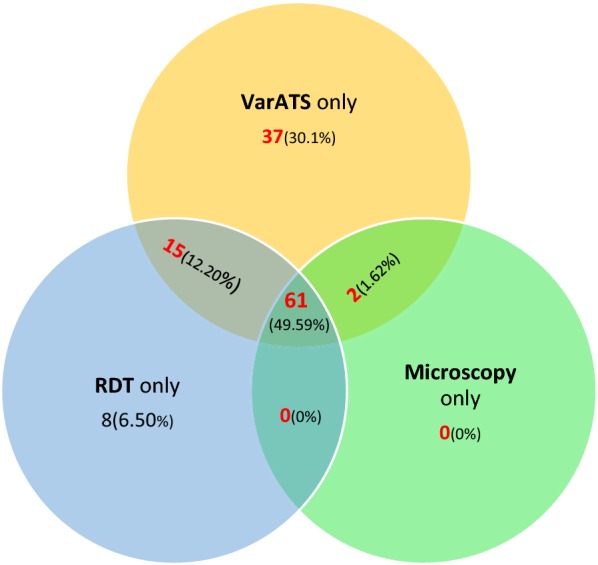
Detection of asymptomatic malaria by RDT, microscopy and *var*ATS qPCR

The proportion of positive cases detected by *var*ATS, microscopy, RDT, RDT/microscopy, *var*ATS/microscopy, *var*ATS/RDT, and *var*ATS/RDT/microscopy were compared. All *P. falciparum*-positive cases detected by microscopy were also *var*ATS positive. However, 8 positive diagnoses by RDT were negative by both microscopy and *var*ATS (Additional file 1: Sheet S1). In addition, 37 (30.1%) of *var*ATS-positive individuals were neither detected by microscopy nor RDT (Fig. 3). The measure of agreement of RDT *versus var*ATS results (Kappa = 0.74) was higher than microscopy versus *var*ATS (Kappa = 0.67). When the sensitivity and specificity of RDT and microscopy were benchmarked against *var*ATS (Additional file 2: Tables S4, S5), higher sensitivity of RDT (73.9%) than microscopy (63.0%) was observed. Microscopy (99.5%) was, however, found to be slightly more specific for asymptomatic infections than RDT (97%). *Var*ATS parasite densities below 174.84/µL were undetectable by microscopy. However, the threshold was slightly lower for RDT, which consistently detected parasitaemia beyond 110.38/uL (Additional file 3: Sheet S1).

**Fig. 4:**
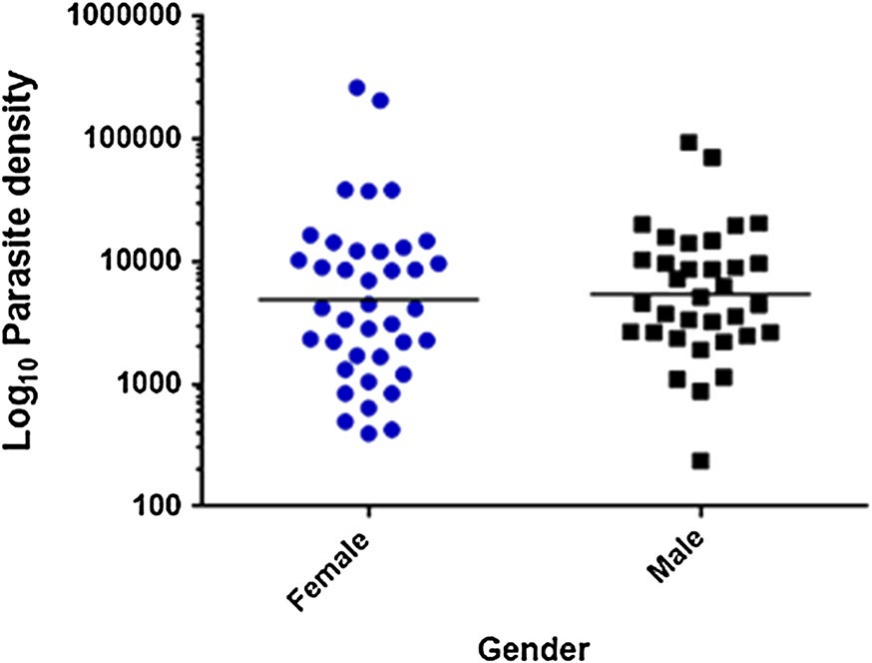
Sex of participants and parasite density by *var*ATS

Furthermore, there was a significant difference in parasite carriage between different age groups (P-value < 0.001; Additional file 2: Table S6). Microscopy and RDT results showed that participants who were positive for asymptomatic malaria were significantly younger than negative individuals (Additional file 2: Tables S7, S8; Table 3). In the *var*ATS group, however, there was no major difference across age groups or gender (Additional file 2: Table S9; Fig. 4).

**Table 3:** Comparison of mean age of study participants

	Mean age ± SD (years)	F	P-value
RDT		21.099	< 0.001
Pos	14.95 ± 14.45		
Neg	26.63 ± 22.76		
Microscopy		11.313	0.001
Pos	16.11 ± 15.45		
Neg	25.39 ± 22.41		
*var*ATS qPCR		6.534	0.011
Pos	19.25 ± 18.50		
Neg	25.51 ± 22.54

## Discussion

Sub-clinical malaria has been associated with mild anaemia [29] and adverse effects in pregnant women [30]. Even more dire is the public health consequence of low-density infections [31]. Accurate prevalence estimates are important for systematic identification and treatment of individuals with asymptomatic falciparum malaria, as part of an intervention strategy, to reduce transmission of the disease. In this study, *var*ATS was used to detect asymptomatic infections among sub-urban settlers in Lagos, Nigeria. The prevalence of asymptomatic malaria reported was high at 35.4%. Although microscopy and *var*ATS diagnosis shared a fairly modest level of agreement, *var*ATS analysis showed that sub-microscopic infections were present in an additional 10% of the population. The lower rate of microscopic detection observed here compared to *var*ATS is consistent with a previous investigation [32].

Analysis of the relationship between RDT and microscopy sensitivity revealed higher numbers of asymptomatic infections detected by RDT relative to microscopy. This challenges existing knowledge, which implies less sensitivity of RDT than microscopy [33] even though this study did not foreclose the possibility of false positive diagnosis by RDT as 8 RDT-positive diagnoses, that were neither microscopy nor *var*ATS positive, were reported. Nonetheless, the RDT detection limit (110.38/µL), benchmarked against *var*ATS in this study, is consistent with the assertion that the detection limit of RDTs is typically around 100 parasites/µL [33]. Meanwhile, the high proportion of sub-microscopic infections, as reported here, provides evidence of ongoing transmission of malaria. This may suggest that the integrated control approach adopted by the Lagos State Malaria Research, Technical and Advisory Committee (LASMARTAC) has not effectively reduced malaria transmission in the community. As asymptomatic infections are enough to restart transmission [34, 35], an intensified approach that will incorporate molecular diagnostics to target, treat and follow up sub-microscopic infections is advocated.

Microscopy and RDT analyses showed that participants who were positive for asymptomatic malaria were significantly younger than those who tested negative. In the *var*ATS group, however, there was no major difference across age groups. This provides additional evidence of the effectiveness of *var*ATS to detect malaria parasites across all age groups. Older individuals are expected to acquire immunity from several episodes of malaria during the early part of their lives [36, 37], hence a higher prevalence of sub-clinical infections should be expected with increasing age. However, the report from this study did not conform to this convention. Specifically, a higher frequency of asymptomatic infections was observed in the 6–14 years group (school-age children) than in individuals in older adults. This corroborates previous findings that revealed high prevalence of asymptomatic parasitaemia in individuals aged 5 to 15 years [38–40]. A plausible interpretation of this finding is that school-age children are possibly more exposed to infection, and they may build up immunity against clinical malaria in the process. Walldorf et al. [40] reported that school-age children sleep under nets less often than any other age group. This lack of utilization of preventive measures and engagement in outdoor activities may partly explain increased prevalence in this group of children. National malaria control programmes should pay more attention to malaria in school-age children. Integration of malaria intervention strategies with other school-based programmes is recommended.

Meanwhile, there could be other factors contributing to the prevalence of sub-microscopic infections that were not described in this study. Treatment inefficacy, for instance, may influence residual carriage of infections [41, 42]. Oyebola et al. [43] reported that artemisinin-based treatment of clinical infections led to the persistence of sub-microscopic parasitaemia. Detailed longitudinal data from drug therapeutic efficacy trials will be required to clarify this observation. Another limitation of this study is that the *var*ATS PCR technique has only been developed for *P. falciparum* diagnosis [18]. Therefore, there is a high chance of non-detection of other malaria species. Moreover, it is important to consider cross-sectional surveillance of ultra-low-density infections for daily fluctuations in parasite density and virulence in subsequent studies. A longitudinal study that effectively measures parasite kinetics, gametocyte production and transmission potential of low-density infections will be useful for malaria control in Nigeria. If provisions are made to subsidize operational costs of *var*ATS, the deployment of the molecular tool in field diagnosis will facilitate malaria elimination.

## Conclusions

This study revealed high cases of asymptomatic malaria in the study population, with *var*ATS detecting additional sub-microscopic infections. The reports suggested that older children were at increased risk of asymptomatic malaria in Bayeku community. A large-scale screening to identify more hotspots of asymptomatic parasite reservoirs in the country is recommended.

## Supplementary information

**Additional file 1.** Calculation of *var*ATS qPCR standards.

**Additional file 2.** Additional tables.

**Additional file 3.** *var*ATS/microscopy vs *var*ATS/RDT raw values.

## Data Availability

The datasets supporting the conclusions of this article are included within the article and its additional files.

## Abbreviations

ATS: acidic terminal sequence
LASMARTAC: Lagos State Malaria Research, Technical and Advisory Committee
PCR: polymerase chain reaction
RDT: rapid diagnostic test
WBC: white blood cells
