# Supplementary material for: High cases of submicroscopic Plasmodium falciparum infections in a suburban population of Lagos, Nigeria
Source: Malar J. 2019 Dec 19;18:433. doi: 10.1186/s12936-019-3073-7 (PMC6924037; doi:10.1186/s12936-019-3073-7)
Supplement: Supplementary file 2 — Additional file 2. Additional tables. [file 12936_2019_3073_MOESM2_ESM.docx]

**Additional Table S1: Diagnosis of asymptomatic malaria by RDT, microscopy and *var*ATS qPCR**

|  | | RDT | | Microscopy | | *var*ATS qPCR | | Total |
| --- | --- | --- | --- | --- | --- | --- | --- | --- |
|  |  | Positive | Negative | Positive | Negative | Positive | Negative |  |
| Sex | Male | 45(39.1%) | 70(60.9%) | 37(32.2%) | 78(67.8%) | 54(47%) | 61(53%) | 115(100%) |
|  | Female | 49(24.2%) | 152(75.6%) | 39(19.4%) | 162(80.6%) | 65(32.3%) | 136(67.7%) | 201(100%) |
| Total | | 94(29.7%) | 222(70.3%) | 76(24.1%) | 240(75.9%) | 119(37.7%) | 197(62.3%) | 316(100%) |

**Additional Table S2: Geometric mean of parasite density by *var*ATS counts between sexes**

| Sex | | Parasite density | *var*ATS |
| --- | --- | --- | --- |
| Male | N | 34 | 54 |
|  | Geometric Mean | 5409.86 | 133.5959 |
|  | Minimum | 238 | .01 |
|  | Maximum | 93636 | 988047079100.00 |
| Female | N | 39 | 65 |
|  | Geometric Mean | 4880.92 | 87.9310 |
|  | Minimum | 396 | .01 |
|  | Maximum | 261000 | 5125574889800.00 |
| Total | N | 73 | 119 |
|  | Geometric Mean | 5120.52 | 106.3094 |
|  | Minimum | 238 | .01 |
|  | Maximum | 261000 | 5125574889800.00 |

**Additional Table S3: Geometric mean of parasite density by *var*ATS counts across different age groups**

| Age group | | *var*ATS |
| --- | --- | --- |
| 1-5 | *N | 12 |
|  | Geometric Mean | 3566.28 |
|  | Minimum | 425 |
|  | Maximum | 93636 |
| 6-14 | N | 39 |
|  | Geometric Mean | 6688.91 |
|  | Minimum | 238 |
|  | Maximum | 204190 |
| >14 | N | 22 |
|  | Geometric Mean | 3884.13 |
|  | Minimum | 838 |
|  | Maximum | 261000 |
| Total | N | 73 |
|  | Geometric Mean | 5120.52 |
|  | Minimum | 238 |
|  | Maximum | 261000 |

*N = Number of participants screened

**Additional Table S4: Sensitivity/specificity of RDT vs *var*ATS PCR**

|  | | | *var*ATS PCR | | Total |
| --- | --- | --- | --- | --- | --- |
|  |  |  | Positive | Negative |  |
| RDT | Positive | Count | 88 | 6 | 94 |
|  |  | % within RDT | 93.6% | 6.4% | 100.0% |
|  |  | % within *var*ATS PCR | *73.9% | 3.0% | 29.7% |
|  | Negative | Count | 31 | 191 | 222 |
|  |  | % within RDT | 14.0% | 86.0% | 100.0% |
|  |  | % within *var*ATS PCR | 26.1% | **97.0% | 70.3% |
| Total | | Count | 119 | 197 | 316 |
|  |  | % within RDT | 37.7% | 62.3% | 100.0% |
|  |  | % within *var*ATS PCR | 100.0% | 100.0% | 100.0% |
| Pearson Chi-Square | | 178.468 | | | |
| P-value | | .000 | | | |

*Sensitivity **Specificity

**Additional Table S5: Sensitivity/specificity of microscopy vs *var*ATS PCR**

|  | | | PCR | | Total |
| --- | --- | --- | --- | --- | --- |
|  |  |  | Positive | Negative |  |
| Microscopy | Positive | Count | 75 | 1 | 76 |
|  |  | % within Microscopy | 98.7% | 1.3% | 100.0% |
|  |  | % within *var*ATS qPCR | *63.0% | .5% | 24.1% |
|  | Negative | Count | 44 | 196 | 240 |
|  |  | % within Microscopy | 18.3% | 81.7% | 100.0% |
|  |  | % within *var*ATS qPCR | 37.0% | **99.5% | 75.9% |
| Total | | Count | 119 | 197 | 316 |
|  |  | % within Microscopy | 37.7% | 62.3% | 100.0% |
|  |  | % within *var*ATS PCR | 100.0% | 100.0% | 100.0% |
| Pearson Chi-Square | | | 158.738 | | |
| P-value | | | <.001 | | |

*Sensitivity **Specificity

**Additional Table S6: Age groups and asexual parasitaemia (*var*ATS)**

|  | | Asexual parasitaemia |
| --- | --- | --- |
|  |  | Positive |
| Age group | 1-5 | 12(17.9%) |
|  | 6-14 | 40(41.7%) |
|  | >14 | 22(14.4%) |
| Total | | 74(23.4%) |
| Pearson Chi-Square | | 25.930 |
| P-value | | 0.0311 |

**Additional Table S7: Age vs RDT Positivity**

| mRDT | Mean Age | Std. Deviation | Number | Minimum | Maximum |
| --- | --- | --- | --- | --- | --- |
| Positive | 14.952 | 14.4457 | 94 | 1.0 | 67.0 |
| Negative | 26.625 | 22.7629 | 222 | 1.0 | 100.0 |
| Total | 23.153 | 21.3003 | 316 | 1.0 | 100.0 |

P = 0.0000063

**Additional Table S8: Age vs microscopy Positivity**

| Microscopy | Mean Age | Std. Deviation | Number | Minimum | Maximum |
| --- | --- | --- | --- | --- | --- |
| Positive | 16.105 | 15.4493 | 76 | 1.0 | 67.0 |
| Negative | 25.385 | 22.4116 | 240 | 1.0 | 100.0 |
| Total | 23.153 | 21.3003 | 316 | 1.0 | 100.0 |

P = 0.0086

**Additional Table S9: Age vs *var*ATS qPCR**

| *var*ATS PCR | Mean Age | Std. Deviation | N | Minimum | Maximum |
| --- | --- | --- | --- | --- | --- |
| Positive | 19.246 | 18.5021 | 119 | 1.0 | 85.0 |
| Negative | 25.513 | 22.5435 | 197 | 1.0 | 100.0 |
| Total | 23.153 | 21.3003 | 316 | 1.0 | 100.0 |

P = 0.011
